# The Probiotic Effects of the *Saccharomyces cerevisiae* 28-7 Strain Isolated from Nuruk in a DSS-Induced Colitis Mouse Model

**DOI:** 10.4014/jmb.2206.06035

**Published:** 2022-06-30

**Authors:** Jang Eun Lee, Eunjung Lee

**Affiliations:** 1 Reserch Group of Traditional Food, Korea Food Research Institute, Wanju-gun, Jeollabuk-do 55365, Republic of Korea; 2 Department of Food Biotechnology, Korea University of Science and Technology, Daejeon 34113, Republic of Korea

**Keywords:** Probiotic yeast, *Saccharomyces cerevisiae* 28-7, *Saccharomyces boulardii*, nuruk, colitis

## Abstract

Probiotics are microorganisms that can benefit host health when ingested in a live state, and lactic acid bacteria are the most common type. Among fungi, *Saccharomyces boulardii* (SB) is the only strain known to have a probiotic function with beneficial effects on colitis; however, information on other probiotic yeast strains is limited. Therefore, this study aimed to discover yeast strains expressing intestinal anti-inflammatory activities by exhibiting probiotic properties in dextran sodium sulfate (DSS)-induced colitis mice model. Nuruk (Korean traditional fermentation starter) containing various microbial strains was used as a source for yeast strains, and *S. cerevisiae* 28-7 (SC28-7) strain was selected with in vitro and in vivo characteristics to enable survival in the intestines. After 14 days of pretreatment with the yeast strains, DSS was co-administered for six days to induce colitis in mice. The results revealed that the disease activity index score was lowered by SC28-7 treatment compared to the DSS group, and the colon length and weight/length ratio were recovered in a pattern similar to that of the normal group. SC28-7 administration significantly reduced the secretion of pro-inflammatory cytokines in the serum and modified the mRNA expression of inflammatory cytokines (interleukin-1β, transforming growth factor-β, and interferon-γ) and proteins involved in gut barrier functions (mucin 2, mucin 3, zonula occludens-1, and occludin) in colon tissues. These results indicate that SC28-7 attenuates DSS-induced colon damage and inflammation, supporting its future use as a probiotic yeast for treating and preventing intestinal inflammatory diseases such as inflammatory bowel disease.

## Introduction

Probiotics are living organisms that may benefit the intestinal health of hosts and are mainly comprised of lactic acid bacteria such as *lactobacillus*, *Bifidobacterium*, and *Streptococcus* [1]. However, eukaryotic microorganisms, especially *Saccharomyces cerevisiae*, have been recently studied for their probiotic function; therefore, yeast is widely used as a treatment strategy for relieving intestinal imbalance. The most widely known probiotic yeast is *S. cerevisiae* *var. boulardii* (*S. boulardii*) [2]; however, some yeast genera, such as *Debaryomyces*, *Wickerhamomyces*, *Kluyveromyces* and *Torulaspora* also have probiotic potentials [3]. The yeast probiotics are different from bacterial probiotics in terms of the physiological structure, size, the survival rate in the gut, and antibiotic resistance; therefore, the latest probiotic market is growing with both lactic acid bacteria and yeast strains [4]. Owing to these interests in probiotic yeast, several studies have been conducted on *S. boulardii*. The clinical effect of ingesting live *S. boulardii* cells has been demonstrated in food-borne gastroenteritis caused by *Clostridium vibrio* and chronic diarrhea caused by pathogenic microbial infection [5]. *S. boulardii* alleviates most cases of infectious diarrhea by directly impacting pathogens or toxins and the host infection-induced signaling cascades and influencing the innate and adaptive immune systems [6]. In addition, as the inhibitory effect of *Helicobacter* on gastric lymphoid follicles has been revealed, the supplementation of this yeast strain has been used to treat gastritis *H. pylori* and mitigate symptoms [7]. Thus, *S. boulardii* has been developed as a candidate for this therapeutic approach using a limited number of *S. cerevisiae* strains genetically similar to it. This study isolated *S. cerevisiae* strains with probiotic properties from the traditional Korean nuruk, a starter for brewing alcoholic beverages. Nuruk is fermented by naturally inoculated microorganisms and contains diverse environmental microorganisms; therefore, it can be utilized to discover various characteristics and potential probiotic strains. As various microorganisms are abundantly found in nuruk, they can be widely utilized as raw materials for fermented foods and as agents for isolating useful microbial resources. Previous studies have shown that *Makgeolli* brewed with nuruk or a yeast strain isolated from nuruk can alleviate disruption of gut microbiota homeostasis or systemic inflammation [8, 9]. This study aimed to extract probiotic *S. cerevisiae* from nuruk and evaluate its probiotic properties to alleviate intestinal inflammation in mice colitis model, highlighting the application of *S. cerevisiae* derived from nuruk as a probiotic yeast supplement for gut health.

## Materials and Methods

### Isolation and Selection of Probiotic Yeast

*S. cerevisiae* were isolated from the traditional Korean nuruk and stored according to the manufacturing method of ancient literature in previous studies [10]. Over 200 yeast colonies were cultured in potato dextrose agar (PDA; Becton Dickinson and Company, USA) media at 25°C for 48 h and stored at 4°C before analytical procedures. To investigate the potential properties of yeast probiotics, galactose assimilation, acid tolerance, and high-temperature resistance were assessed. For galactose assimilation analysis, 100 μl of each yeast culture was inoculated into 10 ml of yeast nitrogen base without amino acids (YNB W/O AA) (Difco 291940, Difco, USA) media containing 10% galactose, and the carbon dioxide generation in the Durham tube was examined at 25°C incubation. In addition, Yeast and mold (YM) media (BD-Difco, USA) adjusted to pH 2.0 with 0.1 M hydrochloric acid (HCl) and 37°C of cultivation temperature were used to determine the acid and high-temperature resistance of isolated yeast strains. Through these processes, the *S. cerevisiae* 28-7 (SC28-7) strain was selected as a candidate probiotic yeast and deposited at the Korean Culture Center of Microorganisms (KFCC11825P).

### Identification of SC28-7 strain

The SC28-7 strain was identified by 18S ribosomal RNA (rRNA) sequencing using ITS1 (5′-TCCG TAGGTGAACCTGCGG-3′) and ITS4 (5′-TCCTCCGCTT ATTGATATGC-3′) primers. After purification of the amplified products, sequencing was performed using an ABI PRISM 3780XL DNA analyzer (Applied Biosystems, USA) and the basic local alignment search tool (BLAST) program (http://www.ncbi.nlm.nih.gov).

### Yeast Cell Sample Preparation

Live yeast cells were prepared for oral administration using the following procedure. SB yeast strain was isolated from a commercially available Bioflor (Laboratories BIOCODEX, France) *Saccharomyces boulardii* National Collection of Microorganism Culture [CNCM] I-745 strain as a positive control yeast strain. *S. cerevisiae* S288C (SC288c) strains were purchased from the American Type Culture Collection (ATCC, USA). Each yeast strain was cultured in potato dextrose broth (PDB) medium at 28°C for 60 h and collected by centrifugation (6,000 ×*g*, 5 min, 4°C). The collected cell pellets were diluted with 20% glycerol in 0.85% sodium chloride solution to a concentration of 1.0×10^8^ CFU/ml viable cells and stored frozen at -80°C until administration. The frozen yeast cells were thawed at 25°C shortly before oral gavage, washed twice with saline, and then re-suspended in saline for oral administration to mice.

### Animals and Experimental Design

Animal experiments were performed with the approval of the Animal Experimental Ethics Committee of the Korea Food Research Institute (IACUC approval number: KFRI-M-19009). Six-week-old male C57BL/6 mice were purchased (Orient Bio, Korea) and housed in a facility controlled with temperature (22 ± 2°C), humidity (55± 5%), and a dark-light cycle of 12 h. During the experiment, a standard diet (Harlan 2019S; Harlan Teklad, USA) and drinking water were provided *ad libitum*. Mice were randomly divided into five groups (eight mice per group) after a one-week adaptation period and confirming no significant difference in weight between the groups. The five groups were the normal group (Cont), the dextran sulfate sodium salt-induced colitis group (DSS; 36,000-50,000 DA; MP Biomedicals, USA), the DSS-induced colitis after SC28-7 strain administration experimental group (DSS+SC28-7), SB strain as a positive control (DSS+SB), and SC288c as a negative control (DSS+SC288c). After the one-week adaptation period, mice were orally administered live yeast cells daily at a concentration of 1.0×10^8^ suspended in 0.2 ml of saline. The Cont and DSS groups were only orally administered vehicle (saline). After two weeks of administration, 3% DSS was mixed with drinking water and administered for six days, and the disease activity index (DAI) was scored and recorded [11]. Oral administration of yeast cells and the vehicle was continued during DSS treatment. On the sixth day, drinking water was replaced with normal water; after three days, the mice were anesthetized and sacrificed after fasting for 15 h. After blood collection, the colon was removed, and the length and weight were measured. Serum and colon samples were stored at -80°C until RNA analysis and cytokine array experiments were conducted. Cecal contents were collected for the quantification of live yeast cells.

### Quantification of Live Yeast Cells

Live yeast cells in the colon were quantified as previously described [9, 12]. Total RNA of the cecal contents was prepared using the RNeasy Power Microbiome Kit (Qiagen, USA), and quantification was performed using a μDrop plate of Varioskan Flash (Thermo, USA). Total RNA (1 μg) was used to synthesize complementary DNA (cDNA) using the iScript cDNA Synthesis Kit (Bio-Rad, USA). For the measurement of the standard curve, a culture solution of 1 × 10^9^ SC288C yeast cells was prepared, and its total RNA was extracted in the same manner, employed to synthesize cDNA. Yeast-F (5′-GAGTCGAGTTGTTTGGGAATGC-3′) and Yeast-R (5′-TCTCTT TCCAAAGTTCTTTTCATCTTT-3′) were used as primers for the quantification of yeast cells, and the primers SC-F (5'-GAAAACTCCACAGTGTGTTG-3') and SC-R (5'-GCTTAAGTGCGCGGTCTTG-3') for quantification of Saccharomyces cells were used to perform real-time polymerase chain reaction (RT-PCR) with the StepOnePlus RT-PCR system (Applied Biosystems, USA) and Fast SYBR Green Master Mix (Applied Biosystems). All procedures were performed according to the manufacturer's instructions. The standard curve of SC288c cells was expressed as log10 of yeast per gram of cecal content, repeated three or more times, and expressed as mean value and standard deviation.

### Inflammatory Cytokine Antibody Array

Blood samples obtained from the mice were transferred into serum-separating tubes (SST; BD Biosciences, USA) and allowed to clot for 30 min at 25°C before centrifuging for 10 min at 2,000 ×*g*. Serum cytokine levels were analyzed using a mouse cytokine antibody array kit (ART006, R&D Systems, USA) with 0.1 ml serum samples, according to the manufacturer's instructions. Visualization and quantification of the array membranes were conducted using Alliance Mini HD9 (UVITEC, UK) and ImageJ software (1.49v, NIH, USA), respectively. All quantified data were normalized to the pixel intensity of the reference spot on each membrane according to the manufacturer's instructions.

### Analysis of Gene Expression by Quantitative RT-PCR

Total RNA (1 μg) from mice colon samples was used to synthesize cDNA using an iScript cDNA synthesis kit (Bio-Rad). Quantitative RT-PCR was performed using a StepOnePlus instrument (Applied Biosystems) and a Fast SYBR Green Master Mix kit (Life Technologies). Relative mRNA expression was determined using specific primers (Table 1) and normalized to that of β-actin.

### Statistical Analysis

All graphs are expressed as mean ± standard error (S.E.) and analyzed by one-way analysis of variance (ANOVA), followed by Dunnett’s post-hoc test. A *p*-value < 0.05 was considered statistically significant (#, *p* < 0.05; ##, *p* < 0.01; ###, *p* < 0.001 between Cont and DSS; *, *p* < 0.05; **, *p* < 0.01; ***, *p* < 0.001 vs. DSS). All statistical analyses were performed using the Prism software (GraphPad Software, USA).

## Results and Discussion

### The Selection and Identification of Probiotic Yeast from Nuruk

**Fig. 1:**
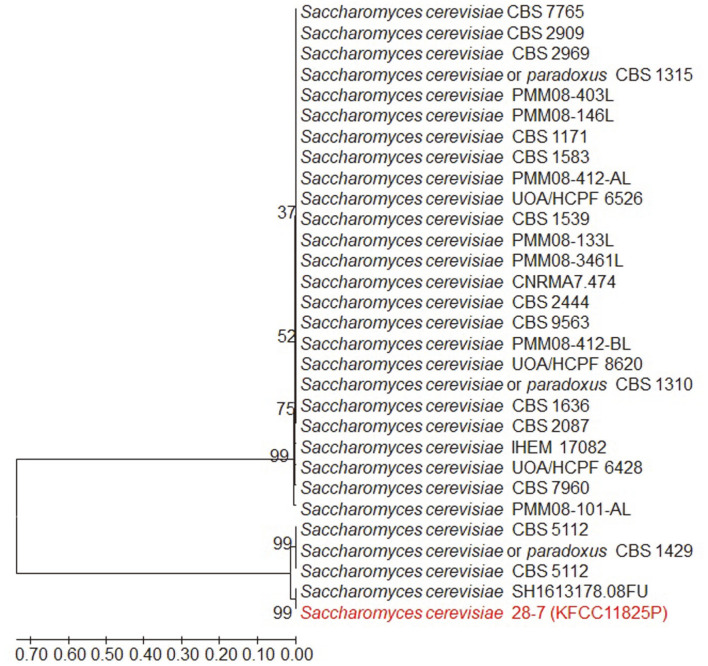
Neighbour-joining tree based on ITS region sequences showing the position of *Saccharomyces cerevisiae* 28-7 strain. Numbers at branch nodes are bootstrap percentage based on 1000 bootstrap resampling. The evolutionary distances were computed using the Tamura 3-parameter method. Bar, 0.1 substitutions per nucleotide position.

The representative characteristics of *S. boulardii*, the probiotic yeast, compared to non-probiotic *S. cerevisiae* are low galactose assimilation, acid, and high-temperature resistance [13]. This study investigated probiotic candidate strains based on these characteristics. According to previous studies, *S. boulardii* could not assimilate galactose as a carbon source despite its related genes [13, 14], and it consumed galactose at a much lower rate than non-probiotic *S. cerevisiae*. The molecular mechanisms underlying inefficient galactose utilization in *S. boulardii* yeast have not been elucidated; however, it has been reported that its low galactose utilization is due to mutation of the Phosphoglucomutase 2 (PGM2) gene [13]. This study selected approximately 20 of 200 probiotic candidate yeast colonies based on galactose assimilation characteristics (data not shown). This screening method has the advantage of being relatively simple and capable of accurately testing strain characteristics. However, additional studies are needed to elucidate the mechanisms of galactose non-fermentation in *S. boulardii* strains and the factors that may indicate high galactose fermentability in some *S. boulardii* strains. From the 20 candidate colonies, SC28-7 strains were ultimately selected through high acid and temperature tolerance analyses (data not shown). The superior survival rate of *S. boulardii* yeast under high pH and temperature conditions is an important factor for yeast survival as live cells in the intestine [15]. A pH of 4.0 has been suggested as a threshold value that does not alter *S. cerevisiae* viability [16]. Additionally, it has been reported that yeast growth was not observed at a pH of 1.5-2.0, indicating that viability at a low pH near 2.0 is crucial to the characteristics of probiotic yeast [17]. This study investigated a few yeast strains that showed high resistance at a pH of 2.0 with high viability at 37°C, and the probiotic yeast strain, SC28-7, was ultimately selected. The phylogenetic tree of this strain is presented in Fig. 1. The *S. boulardii* yeast was previously classified as a different species in the Saccharomyces genus, although it has a very similar genetic homology to *S. cerevisiae*. However, draft genome sequence analysis revealed that the two strains share more than 99% of genomic information, and *S. boulardii* yeast has been classified as a cluster of wine yeast in the phylogenetic tree[18]. Due to the high homology of *S. cerevisiae*, *S. boulardii* yeast is taxonomically classified as *S. cerevisiae*; however, there are significant phenotypic differences between the two strains, such as the probiotic activity. Therefore, *S. cerevisiae* with probiotic functions is precisely specified as *S. cerevisiae* var. *S. boulardii* which is currently used as a probiotic yeast.

### The Alleviating Effect of SC28-7 on DSS-Induced Mice Colitis

**Fig. 2:**
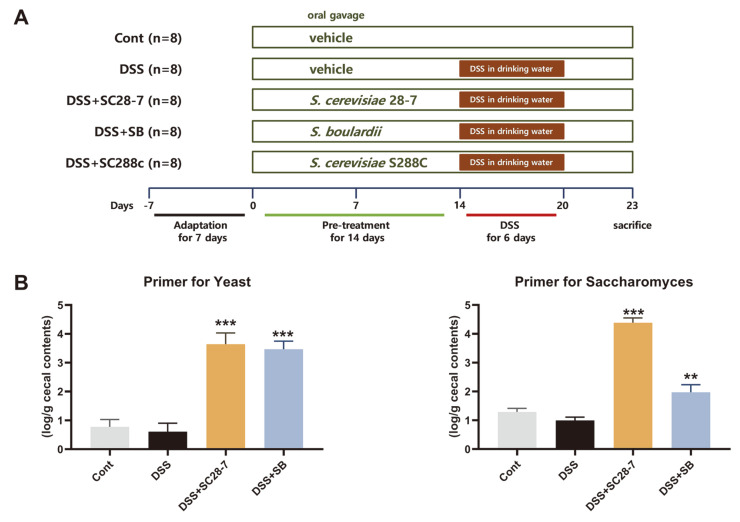
(**A**) Experimental design of dextran sodium sulfate (DSS)-induced colitis mouse models and (**B**) quantification of live yeast cells in cecal contents from mice. The graphs using the primer for the yeast (left) and the specific primer for Saccharomyces (right) are shown as mean ± S.E. determined from eight mice from each group. **p < 0.01, *** *p* < 0.001 vs. DSS.

**Fig. 3:**
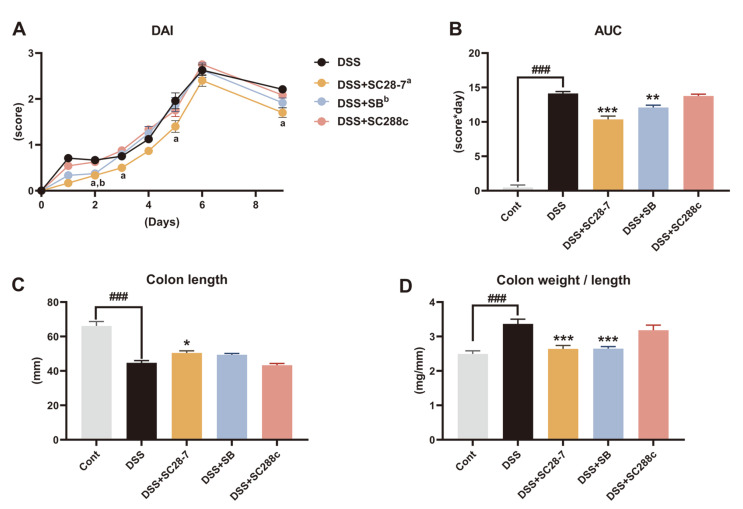
Effects of SC28-7 administration on DSS-induced clinical symptoms of colitis in mice. (**A**) Disease activity index (DAI) is the average of the composite measures of weight loss, stool consistency and bleeding in stool after initiation of DSS treatment. In the graph, “a” represents the statistical significance (*p* < 0.05) of DSS and DSS+SC28-7 on the same day, and “b” means the significant difference (*p* < 0.05) between DSS and DSS+SB. (**B**) Area under curve (AUC) values of DAI for nine days after DSS treatment. (**C**) Changes in colon length. (**D**) Colon weight/length ratio in the DSS-colitis model. The data represent means ± S.E. (*n* = 8 mice/group). ### represents the significant difference between Cont and DSS group (*p* < 0.001). *p < 0.05, **p < 0.01, *** *p* < 0.001 vs. DSS.

The possible probiotic effect of SC28-7 in vivo was examined using DSS-induced colitis mouse model (Fig. 2A). To confirm whether SC28-7 is viable in the gastrointestinal (GI) tract, RNA from the cecal contents was extracted and used to quantify live yeast cells (Fig. 2B). The significant presence of live yeast cells was confirmed in the SC28-7 and DSS+SB groups compared to the Cont and DSS groups that were not administered yeast cells. This result indicated that the additional administration of SC28-7 and SB induced an increase in the number of viable yeast cells in the GI tract. After DSS treatment, the DAI score of the DSS group rose from 0.76 on the first day to 2.71 after the sixth day and was maintained at 2.21 three days after replacement with normal drinking water, confirming that colitis and intestinal inflammation were well induced by DSS treatment (Fig. 3A). In the DSS+SC28-7 group, the DAI score increased from 0.17 on the first day to 2.40 after the sixth day; however, after replacing it with normal drinking water, it reduced to 1.70. To compare and analyze the overall level of the DAI score, the DAI for nine days was calculated and confirmed as an area under the curve (AUC) (Fig. 3B). Compared with the DSS group, the DSS+SB group as a positive control and the DSS+SC28-7 group showed a significant tendency (*p* = 0.003 and *p* < 0.001, respectively) to decrease the AUC, but not the SC288c group (*p* = 0.889). As an indirect indicator for confirming inflammation levels in the colon, the length and weight of the colon were measured, and the ratio was confirmed. The length of the colon in the DSS+SC28-7 group showed a significant increase (Fig. 3C), and it was also confirmed that the weight/length ratio decreased (Fig. 3D) compared to that of the DSS group. Previous studies have demonstrated that an increase in the colonic weight/length ratio is directly associated with the severity of colon damage in this colitis model [19, 20]. In this study, the administration of SC28-7 resulted in a significant decrease in the colon weight/length ratio compared to the DSS-treated group, indicating a beneficial effect of SC28-7. In contrast, SC288c administration did not alter colon length or weight. These results suggested that DSS-induced colitis was not alleviated by yeast administration alone but by the administration of SB and SC28-7 strains.

### SC28-7 Consumption Reduces Systemic Inflammatory Responses Induced by the DSS Treatment in Mice

**Fig. 4:**
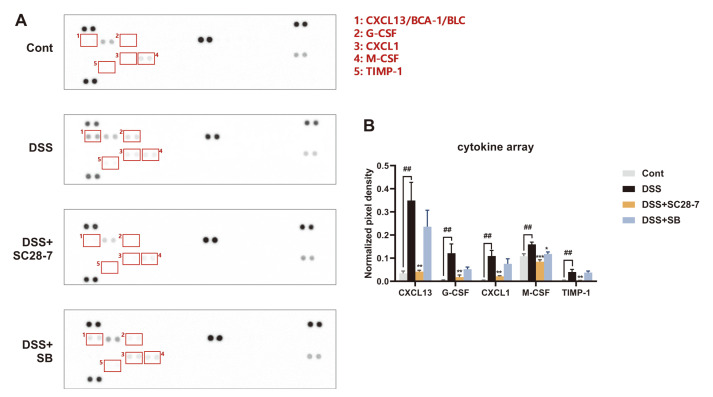
Effects of SC28-7 administration on DSS-induced inflammatory cytokine secretion in serum. (**A**) The representative pictures of the protein blots showing inflammatory cytokine array dots by group (*n* = 4 serum samples/group). The numbered boxes above the blots indicate the proteins with the significant differences between the Cont and DSS groups shown in the upper right corner. (**B**) Quantification of the relative spot intensity normalized to positive control spot of each blot. Quantitative analysis was performed using Image J software with four independent experiments. ##*p* < 0.01 between Cont and DSS, *p < 0.05, **p < 0.01, ***p < 0.001 vs. DSS.

To examine the protective effects of SC28-7 on DSS-induced colitis and systemic inflammation, a cytokine antibody array was conducted using mice serum samples. As shown in Fig. 4A, the representative pictures of the array membrane reacted with mouse serum of each group showed that the expression of five inflammatory cytokines increased with DSS treatment and decreased with SC28-7 administration. Among them, chemokine (C-X-C motif) ligand 13 (CXCL13, labeled 1 in Fig. 4A) showed the most dramatic change after DSS and SC28-7 treatment. It showed an average of ten times higher spot intensity than the Cont group after DSS treatment, and the SC28-7-treated group showed a significant decrease of 88% (Fig. 4B). CXCL13 is also known as a B lymphocyte chemoattractant (BLC) or B cell-attracting chemokine 1 (BCA-1). It has been reported to be upregulated in human and mouse colon cancers [21-23] and in the serum of azoxymethane (AOM)/DSS-induced intestinal colorectal adenocarcinoma model compared to that in the control groups [24]. CXCL13 deficiency in mice showed a protective effect against intestinal injury by AOM/DSS treatment, whereas its activation was associated with inflammatory signaling pathways, such as AKT and nuclear factor kappa B (NF-κB) signaling [24]. In addition, the levels of other pro-inflammatory cytokines (markers) such as granulocyte colony-stimulating factor (G-CSF), CXCL1, and tissue inhibitor matrix metalloproteinase 1 (TIMP-1), were increased by DSS treatment and reduced by only the SC28-7 administered group. Similar to CXCL13, CXCL1 is significantly upregulated in the serum of patients with IBD [25], and targeted treatment of G-CSF induces protective tumor immunity in mouse colorectal cancer [26]. TIMP-1 has also been reported as a novel serum biomarker for colorectal cancer diagnosis [27]. These previous studies suggest that the inflammatory response induced by DSS can be alleviated through SC28-7 administration, normalizing the abnormal immune response in the colon.

### SC28-7 Administration Prevents DSS-Induced Colonic Inflammation and Barrier Dysfunction in Mice

**Fig. 5:**
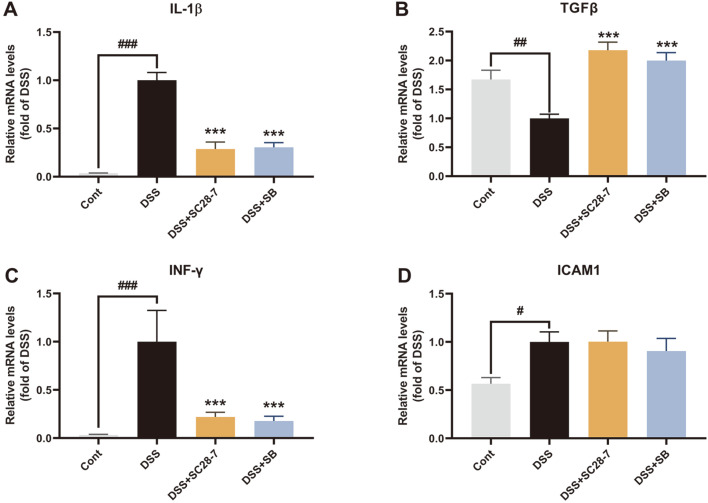
SC28-7 administration influences colonic inflammatory signaling in DSS-induced colitis mice. The mRNA levels of cytokines interleukin (IL)-1β (**A**), transforming growth factor-β (TGFβ) (**B**), interferon (INF)-γ(**C**), and intercellular adhesion molecule (ICAM) 1 (**D**) in colon tissues were quantified by real-time PCR. Fold changes are expressed as means ± S.E. (*n* = 8 each group). #*p* < 0.05, ##*p* < 0.01, and ###*p* < 0.001 between Cont and DSS. *** *p* < 0.001 vs. DSS.

**Fig. 6:**
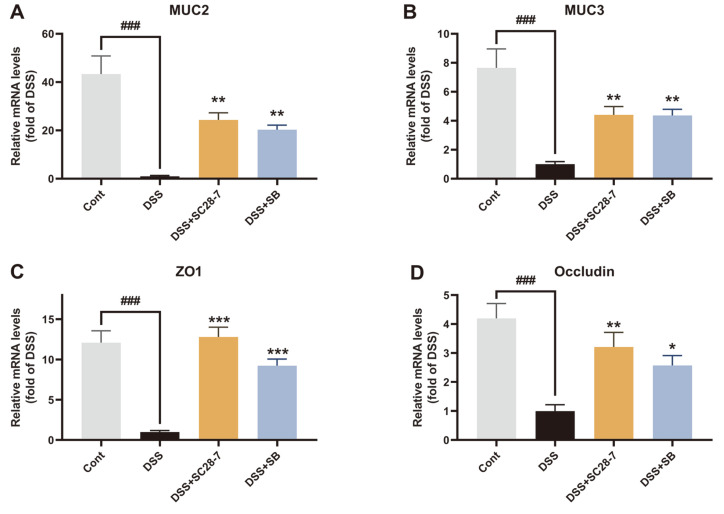
The effects of SC28-7 administration on DSS-colitis on mRNA expression of the intestinal barrier function. The colon tissues from mice of each group (*n* = 8) were used for quantification of the mRNA levels of mucin (MUC)2 (**A**), MUC3 (**B**), ZO1 (**C**), and Occludin (**D**). Fold changes are expressed as means ± S.E. (*n* = 8 each group). ###*p* < 0.001 between Cont and DSS. *p < 0.05, **p < 0.01, *** *p* < 0.001 vs. DSS.

To further determine whether SC28-7 could alleviate colonic inflammation and dysfunction of the colonic mucosa induced by DSS, inflammatory cytokines for biochemical analysis of colon tissues were investigated. The upregulation of the pro-inflammatory cytokines interleukin (IL)-1β and interferon (IFN)-γ by DSS treatment was significantly reduced by SC28-7 administration (Figs. 5A and 5C). Treatment with the SC28-7 strain restored the mRNA expression levels of colonic transforming growth factor (TGF)-β to a level similar to that of non-colitis mice (Fig. 5B). TGF-β is a cytokine that exhibits anti-inflammatory and pro-inflammatory properties depending on the environment of the colonic inflammatory process [28]. It exhibits dual expression tendencies to either decrease or increase depending on the degree and condition of inflammation, indicating that it is important to maintain its normal level for immune homeostasis [20, 29, 30]. These results confirm that SC28-7 has an intestinal anti-inflammatory effect and can induce positive changes in the DSS-induced altered colonic immune response. However, no significant change was observed in the expression of intercellular adhesion molecule (ICAM)-1, consistent with the results of a previous study that evaluated the probiotic effect of SB [20]. Additionally, changes in the expression levels of proteins involved in epithelial integrity and barrier function were determined to explore the effect of SC28-7 administration on barrier dysfunction caused by DSS-induced colonic inflammation. Compared with the Cont group, mucins (MUC2 and MUC3) were significantly downregulated in the DSS-treated mice, and these reductions were recovered by SC 28-7 administration (Figs. 6A and 6B). In addition, the mRNA expression levels of zonula occludens-1 (ZO-1) and occludin, which are involved in tight junctions, were significantly reduced with DSS treatment; however, these reductions were reversed by SC28-7 treatment (Figs. 6C and 6D). These results suggest that the immune response and mucosal dysfunction in mice colitis were restored by SC28-7 administration, supporting its beneficial probiotic effects.

This study established that live SC28-7 administration alleviated DSS-induced colitis by reducing systemic and colonic inflammation. SB strain (*S. boulardii* CNCM I-745), a commercially available probiotic yeast demonstrated to be involved in intestinal immunity and inflammation [11, 31], was used as the positive control. Although a wide range of research has been conducted to distinguish between *S. cerevisiae* and *S. boulardii*, a clear classification criterion has not yet been established, and *S. boulardii* is still classified as *S. cerevisiae* (*S. cerevisiae* HANSEN CBS 5926). The results of this study also confirmed that the SC28-7 strain showed similar and superior probiotic effects to SB. The SC28-7 strain was initially isolated from nuruk for brewing owing to its ability to produce alcohol (data not shown). However, our results suggest that *S. cerevisiae* also has a probiotic effect similar to that of *S. boulardii* depending on the strain. Collectively, our data confirmed that the SC28-7 strain isolated from nuruk alleviates DSS-induced colitis by improving intestinal immune homeostasis and function and reducing systemic and colonic inflammation, suggesting the possibility of developing it into a probiotic yeast. Further research to explore the specific mechanism of action of SC28-7 is necessary.

## Figures and Tables

**Table 1:** Primers used in this study and amplicon size.

Gene symbol	Forward	Reverse	Size (bp)
IL-1β	TCGCTCAGGGTCACAAGAAA	CATCAGAGGCAAGGAGGAAAAC	73
TGFβ	GCTAATGGTGGACCGCAACAAC	CACTGCTTCCCGAATGTCTGAC	100
INF-γ	CGGCACAGTCATTGAAAGCCTA	GTTGCTGATGGCCTGATTGTC	199
ICAM1	GAGGAGGTGAATGTATAAGTTATG	GGATGTGGAGGAGCAGAG	184
MUC2	GATAGGTGGCAGACAGGAGA	GCTGACGAGTGGTTGGTGAATG	134
MUC3	CGTGGTCAACTGCGAGAATGG	CGGCTCTATCTCTACGCTCTCC	112
ZO1	GGGGCCTACACTGATCAAGA	TGGAGATGAGGCTTCTGCTT	234
Occludin	TACGGAGGTGGCTATGGAG	AGGAAGCGATGAAGCAGAAG	109
β-actin	TGTCCACCTTCCAGCAGATGT	AGCTCAGTAACAGTCCGCCTAGA	101
